# Molecular Identification of *Staphylococcus aureus* in Airway Samples from Children with Cystic Fibrosis

**DOI:** 10.1371/journal.pone.0147643

**Published:** 2016-01-25

**Authors:** Emily J. Johnson, Edith T. Zemanick, Frank J. Accurso, Brandie D. Wagner, Charles E. Robertson, J. Kirk Harris

**Affiliations:** 1 University of Colorado, School of Medicine, Aurora, Colorado, United States of America; 2 Department of Pediatrics, University of Colorado School of Medicine, Aurora, Colorado, United States of America; 3 Department of Biostatistics and Informatics, Colorado School of Public Health, University of Colorado School of Medicine, Aurora, Colorado, United States of America; 4 Division of Infectious Diseases, University of Colorado School of Medicine, Aurora, Colorado, United States of America; Queens University Belfast, IRELAND

## Abstract

**Background:**

*Staphylococcus aureus* is a common and significant pathogen in cystic fibrosis. We sought to determine if quantitative PCR (qPCR) and 16S rRNA gene sequencing could provide a rapid, culture-independent approach to the identification of *S*. *aureus* airway infections.

**Methods:**

We examined the sensitivity and specificity of two qPCR assays, targeting the femA and 16S rRNA gene, using culture as the gold standard. In addition, 16S rRNA gene sequencing to identify *S*. *aureus* directly from airway samples was evaluated. DNA extraction was performed with and without prior enzymatic digestion.

**Results:**

87 samples [42 oropharyngeal (OP) and 45 expectorated sputum (ES)] were analyzed. 59 samples (68%) cultured positive for *S*. *aureus*. Using standard extraction techniques, sequencing had the highest sensitivity for *S*. *aureus* detection (85%), followed by FemA qPCR (52%) and 16SrRNA qPCR (34%). For all assays, sensitivity was higher from ES samples compared to OP swabs. Specificity of the qPCR assays was 100%, but 21.4% for sequencing due to detection of *S*. *aureus* in low relative abundance from culture negative samples. Enzymatic digestion increased the sensitivity of qPCR assays, particularly for OP swabs.

**Conclusion:**

Sequencing had a high sensitivity for *S*. *aureus*, but low specificity. While femA qPCR had higher sensitivity than 16S qPCR for detection of *S*. *aureus*, neither assay was as sensitive as sequencing. The significance of *S*. *aureus* detection with low relative abundance by sequencing in culture-negative specimens is not clear.

## Introduction

Airway infection is a significant contributor to morbidity and mortality in patients with cystic fibrosis (CF) [1]. *Staphylococcus aureus* is one of the predominant airway pathogens in patients with CF [2]. Culture-based techniques for identification of airway pathogens have limitations that include time needed for bacterial growth, interference by antibiotics, or overgrowth by other organisms. Molecular methods of identifying microbes may overcome some of these limitations. Improved identification of pathogens could lead to improved treatment [2].

Quantitative PCR assays targeting the small subunit rRNA (16S rRNA) gene have been used to detect bacteria from airway samples [3–6]. However, previous studies have demonstrated poor sensitivity of this qPCR assay in the identification of *S*. *aureus* in airway samples from CF subjects when compared to culture [6]. 16S rRNA qPCR assays specific for other CF pathogens were more sensitive for the identification of other important species in CF including *P*. *aeruginosa* and *H*. *influenzae*, suggesting that different approaches may be needed for detection of *S*. *aureus* [6]. Problems with staphylococcal DNA extraction may contribute to the poor sensitivity of this assay due to the rigid cell wall of *S*. *aureus*, which may limit the isolation of the DNA extraction. Modified extraction methods using lysostaphin and lysozyme may improve staphylococcal DNA extraction [7]. Another potential reason for poor sensitivity is the gene target used, alternate targets such as the femA gene may offer improved sensitivity.

FemA is an essential gene for peptidoglycan synthesis present in methicillin-susceptible *S*. *aureus* (MSSA) and -resistant *S*. *aureus* (MRSA) [8, 9]. The product of the femA gene is involved in the biosynthesis of the cell wall and is necessary for methicillin resistance [10]. Francois et al [11] developed a qPCR assay relying on the nucleic acid amplification of the femA gene for the detection and identification of *S*. *aureus* from clinical samples. This assay was shown to rapidly and accurately identify *S*. *aureus* from mixed DNA obtained from nasal, inguinal, and wound swabs.

Sequencing of the ribosomal RNA gene followed by bioinformatic identification of bacteria has also been used to identify bacteria from airway samples [3, 12, 13]. With decreasing cost of DNA sequencing and more rapid turn-around, this approach may become more practical in clinical settings.

In order for a molecular-based assay to be useful clinically, the sensitivity and specificity of the assay must be comparable to culture, the gold standard. In the current study, we assessed the sensitivity and specificity of *S*. *aureus* detection using two quantitative PCR assays and sequencing, each before and after enzymatic digestion, compared to traditional culture techniques for analyses of oropharyngeal (OP) and expectorated sputum (ES) samples. Furthermore, we correlated quantitative molecular results with quantitative culture results for sputum.

## Methods

### Ethics Statement

The Colorado Multiple Institutional Review Board approved the study (COMIRB 07–0835). Written informed consent and HIPAA Authorization was obtained from patients or guardians. Written informed assent was obtained for children 10–17 years of age.

### Patient Demographics and Samples

Eighty-seven samples from 65 patients with cystic fibrosis ranging in age from 1.5 to 24 years were analyzed consisting of 45 ES samples and 42 OP samples (Table 1). All expectorated sputum and throat swab specimens were collected as part of standard of care for monitoring bacterial infection during routine patient visits. Clinical culture analysis included blood agar plates, MacConkey agar plates, *Haemophilus* isolation agar, *Burkholderia cepacia* selective agar plates, mannitol salt agar, and inhibitory mold agar plates. After clinical cultures were performed, excess specimen was banked and stored frozen at -80 degrees C for molecular assessment of infection. Sputum was homogenized with 10% dithiothreitol following standard CF culture guidelines. Oropharyngeal swabs were vortexed in 1 mL saline and inoculated onto culture plates [14]. 59 samples (68%) were positive and 28 (32%) were negative for *S*. *aureus* by culture.

**Table 1:** Subject/Sample Characteristics.

**Subject characteristics**	**N = 65**
Female, N (%)	33 (51%)
Genotype, N (%)	
*F508/F508*	33 (51%)
*F508/Other*	24 (37%)
*Other*	8 (12%)
Non-Hispanic White, N (%)	57 (88%)
# subjects w/ > 1 sample, N (%)	16 (25%)
Number of samples per subject, median (range)	1 (1–4)
**Sample Characteristics**	**N = 87**
Age of subject at sample collection, median (range)	12.7 (1.5–23.9)
Negative culture, N (%)	8 (9%)
MSSA positive, N (%)	29 (33%)
MRSA positive, N (%)	27 (31%)

### DNA extraction and quantification

**Fig 1:**
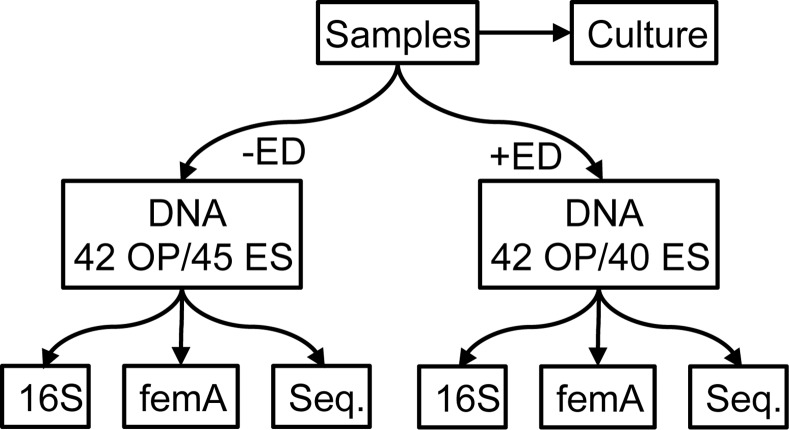
Study Design. Excess specimen collected for standard of care culture surveillance for CF pathogens were frozen at -80C. Aliquots of the thawed specimen (42 OP and 45 ES) were extracted without enzymatic digestion (-ED). A second aliquot was extracted with prior enzymatic digestion (+ED). 5 sputum samples had insufficient quantity for enzymatic digestion. The DNAs obtained were assayed using qPCR that targeted 16S and the femA gene. In addition, sequencing was performed on amplicons generated with pan-bacterial primers targeting the V1/2 region (27F/338R).

DNA extraction was performed using the manufacturer’s protocol for the Qiagen EZ1 Advanced platform. Two aliquots (200 μL) from each specimen were extracted (Fig 1). One was extracted directly without prior enzymatic digestion, and the second was pre-treated based on the protocol developed by Zhao et al [7]. Briefly, samples were mixed with lysostaphin (final concentration 0.18 mg/mL) and lysozyme (3.6 mg/mL) and incubated at 37°C for 30 minutes. Samples were then digested with proteinase K (1.4 mg/mL) and incubated at 65°C for ten minutes. Samples were then incubated at 95°C for 10 minutes. The DNA concentration of each sample was quantified using a Nanodrop Spectrophotometer.

### Quantitative PCR

The quantity of *S*. *aureus* was determined using qPCR assays that target the 16S rRNA gene and the *S*. *aureus* femA gene (Table 2); each assay was performed in triplicate [9, 11]. The femA qPCR assay was modified from Francois et al. to use 400 nM primer concentration (rather than 100 nm in the original description) based on titration of the primer concentration (300 nM to 800 nM) against the cloned femA standards. 400 nM was the lowest concentration where the 100 copy number *S*. *aureus* standard was amplified. Negative controls using sterile water in place of a sample were run in parallel for all experiments. All qPCR data associated with each sample is provided in S1 Table.

**Table 2:** qPCR Primer and Probe Sequences.

Target	Primer	Sequence
*Staphylococcus 16S rRNA*	STPYF	ACGGTCTTGCTGTCACTTATA
	STPYR2	TACACATATGTTCTTCCCTAATAA
*femA-SA*	F femA-SA	TGCCTTTACAGATAGCATGCCA
	R femA-SA	AGTAAGTAAGCAAGCTGCAATGACC
	P femA-SA	TCATTTCACGCAAACTGTTGGCCACTATG

### High-throughput DNA Sequencing

16S Amplicon Library Construction: Bacterial community profiles were determined by broad-range amplification and sequence analysis of 16S rRNA genes following our previously described methods [15, 16]. In brief, amplicons were generated using primers that target approximately 300 base pairs of the V1/2 variable region of the 16S rRNA gene (27F/338R). PCR products were normalized based on agarose gel densitometry, pooled in approximately equal amounts, and the resulting mixture was gel purified and concentrated using a DNA Clean and Concentrator Kit (Zymo, Irvine, CA). Pooled amplicons were quantified using Qubit Fluorometer 2.0 (Invitrogen, Carlsbad, CA). The pool was diluted to 4nM and denatured with 0.2 N NaOH at room temperature. The denatured DNA was diluted to 15pM and spiked with 10% of the Illumina PhiX control DNA prior to loading the sequencer. Illumina paired-end sequencing was performed on the MiSeq platform using a 500 cycle version 2 reagent kit (Illumina, Inc., San Diego, CA)

### Analysis of Illumina Paired-end Reads

Illumina MiSeq paired-end sequences were sorted by sample via barcodes in the paired reads with a python script. Sorted paired end sequence data were deposited in the NCBI Short Read Archive under accession number SRP043334. The sorted paired reads were assembled using phrap [17, 18]. Pairs that did not assemble were discarded. Assembled sequence ends were trimmed over a moving window of 5 nucleotides until average quality met or exceeded 20. Trimmed sequences with more than 1 ambiguity or shorter than 200 nt were discarded. Potential chimeras identified with Uchime (usearch6.0.203_i86linu32) using Schloss Silva reference sequences were removed from subsequent analyses [19, 20]. Assembled sequences were aligned and classified with SINA (1.2.11) using the 629,125 bacterial sequences in Silva 111Ref as a reference configured to yield the Silva taxonomy [21, 22]. Sequences with identical taxonomic assignments were clustered into Operational taxonomic units (OTUs). This process generated 4,397,512 sequences for 168 samples (average size: 26,176 sequences/sample; min: 7,079 max: 63,117). The median Goods coverage score was ≥99.75% at the rarefaction point of 7,079 sequences. The software package Explicet (v2.10.5, www.explicet.org) was used to extract *Staphylococcus* sequence counts for each library [23].

### Identification of *S*. *aureus* among *Staphylococcal* sequences

To identify *S*. *aureus* we employed an ancillary analysis using BLAST to compare the study sequences without enzymatic digestion to the isolate sequences from Silva104 database [24, 25]. BLAST hits were required to have ≥99% sequence identity over ≥95% of the sequence in order to append the Silva binomial name to the RDP taxonomy line [26]. If any of these criteria were not met the sequence retained the genus level classification only.

### Statistical Analysis

Descriptive statistics using means and standard deviations or percentages were used for continuous and categorical variables, respectively. Paired t-tests were used to evaluate whether the DNA concentration between paired samples run with and without enzymatic digestion were significantly different. Assay performance was assessed by calculating sensitivities and specificities using culture as the gold standard. Corresponding 95% confidence intervals were calculated using exact Clopper Pearson type test. Comparisons of sensitivity and specificity across OP and ES samples were performed using a Chi-square test and between methods was performed using McNemar’s test. Spearman’s correlation coefficients were used to associate continuous values for each method. Analyses were performed using SAS version 9.4 software (SAS Institute Inc.: Cary, NC, 2014).

## Results

### DNA Concentration

**Fig 2:**
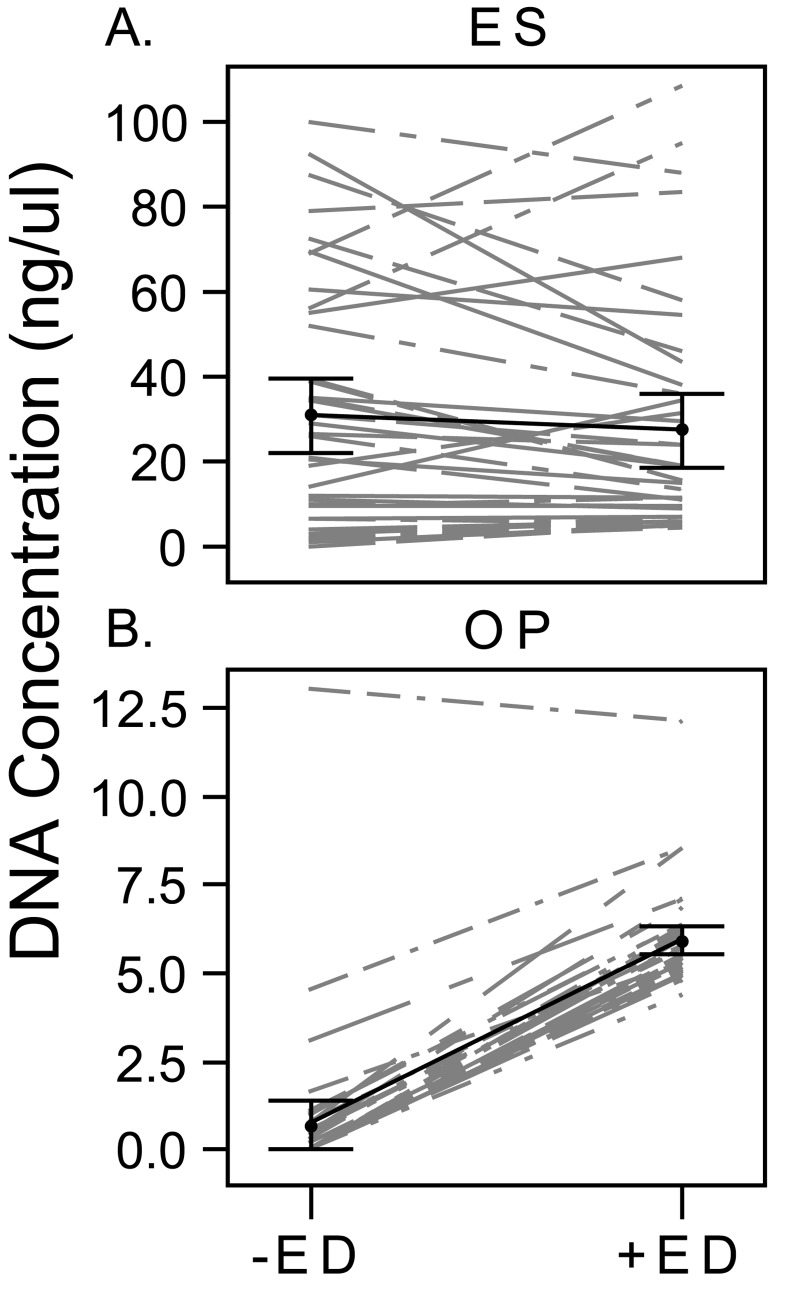
Comparison of DNA Concentration with and without Enzymatic Digestion. DNA concentration is shown for each paired sample of DNA extractions with and without enzymatic digestion for ES (A) and OP (B). The average concentration for all samples with each extraction approach is shown in black with 95% confidence intervals indicated by the whiskers.

For OP samples, DNA concentrations were consistently higher using enzymatic digestion [mean difference in DNA concentration 5.2 ng/μl (SE = 0.2); p<0.01]. For ES samples, there was no significant difference in DNA concentration with or without enzymatic digestion [mean difference in DNA concentration -3.6 ng/μl (SE = 2.6); p = 0.18] (Fig 2).

### Sensitivity and specificity of qPCR assays and sequencing compared to culture

**Fig 3:**
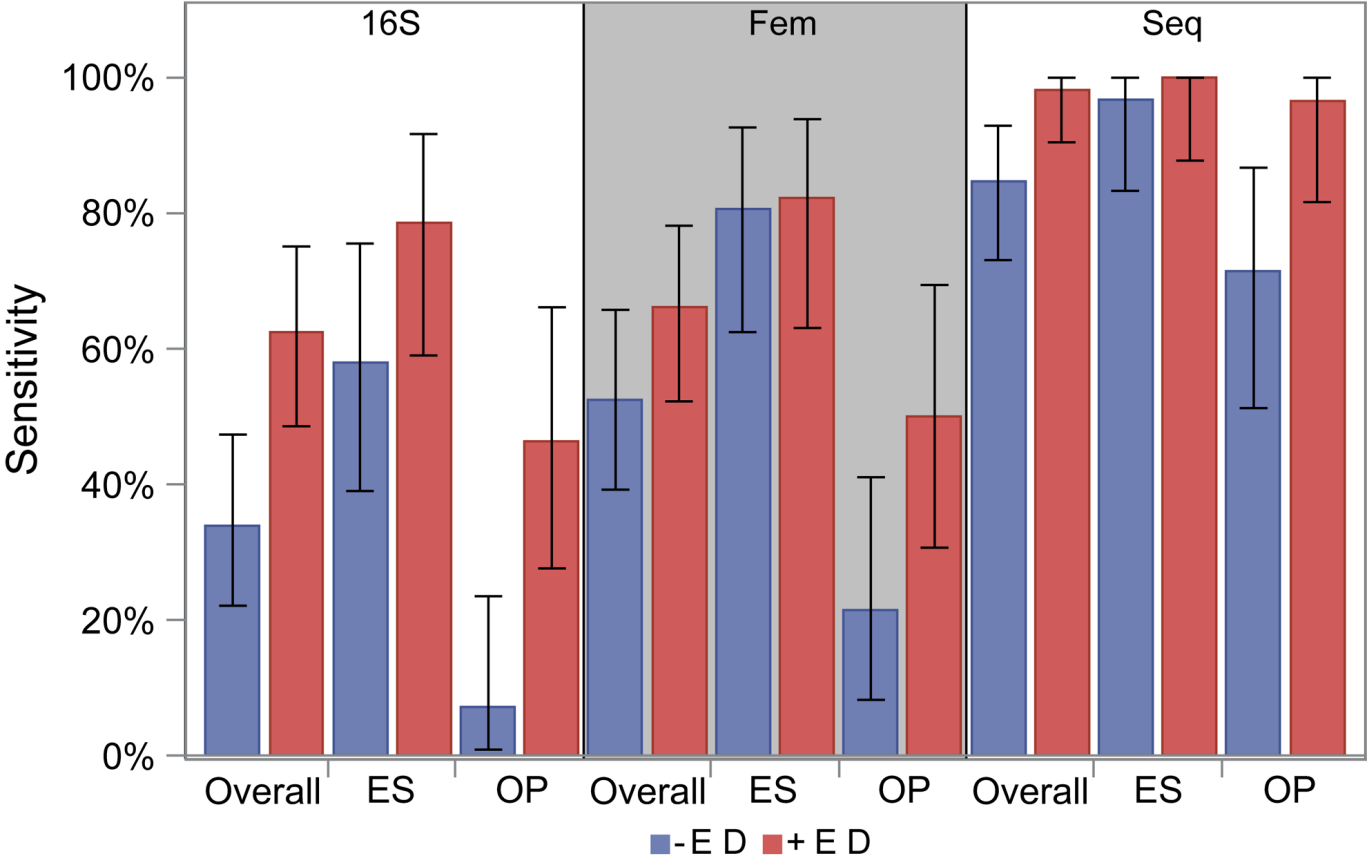
Sensitivity of each molecular assay with and without enzymatic digestion. Sensitivity of qPCR assays compared to standard culture results are given for each assay based on all samples (overall), and by specimen type (ES and OP). Error bars represent 95% confidence intervals. Blue bars are sensitivity without enzymatic digestion, and red bars are with enzymatic digestion.

For both the 16S rRNA assay and the femA assay without enzymatic digestion, OP samples were less sensitive compared to ES samples [16S rRNA: 7.1% (95% CI: 0.1–23.5%) versus 58.1% (95% CI: 39.1–75.5%) femA: 21.4% (95% CI: 8.3–41.0%) versus 80.6% (95% CI: 62.5–92.5%), p < 0.01 for both] (Fig 3). Specificity was 100% for both qPCR assays. Sequencing had higher sensitivity, although similar to qPCR sensitivity from OP samples was lower compared to ES [71.4% (95% CI: 51.3–86.8%) versus 96.8% (95% CI: 83.3–99.9%), p = 0.01]. Specificity of sequencing was low (21.4% (95% CI: 8.3–41.0%) due to detection of *S*. *aureus* from culture negative samples. Culture negative samples had a low median relative abundance of *Staphylococcus* [0.04% (range: 0–0.3%)]. No *Staphylococcus* was detected in negative sterile water controls.

We compared 82 paired samples processed with and without enzymatic digestion. The addition of enzymatic digestion significantly improved the sensitivity of the 16S rRNA assay with OP samples from 7% to 46% (p < 0.01) and ES samples from 58% to 79% (p = 0.01). For the femA assay and sequencing, the addition of the enzymatic digestion significantly improved the sensitivity with OP samples from 21% to 50% and from 71% to 96% (p = 0.01 for both) but not ES samples from 81% to 82% and from 97% to 100% (p = 0.32 and p = 0.99 respectively), although particularly for sequencing, the sensitivity from ES samples was already high with standard processing. Unlike with standard DNA extraction, the sensitivity of the femA assay did not differ from the 16S rRNA assay using enzymatic digestion (p = 0.48). The sensitivity for sequencing from both ES and OP samples was high and did not differ significantly [100% (95% CI: 87.7–100%) versus 96.4% (95% CI: 81.7–99.9%) (Fig 3, p = 0.3)]. The specificity for qPCR assays did not change with enzymatic digestion (100%), and remained low with enzymatic digestion for sequencing [7.7% (95% CI: 0.1–25.1%)].

### Quantitative Comparison of qPCR and sequencing results to culture

**Fig 4:**
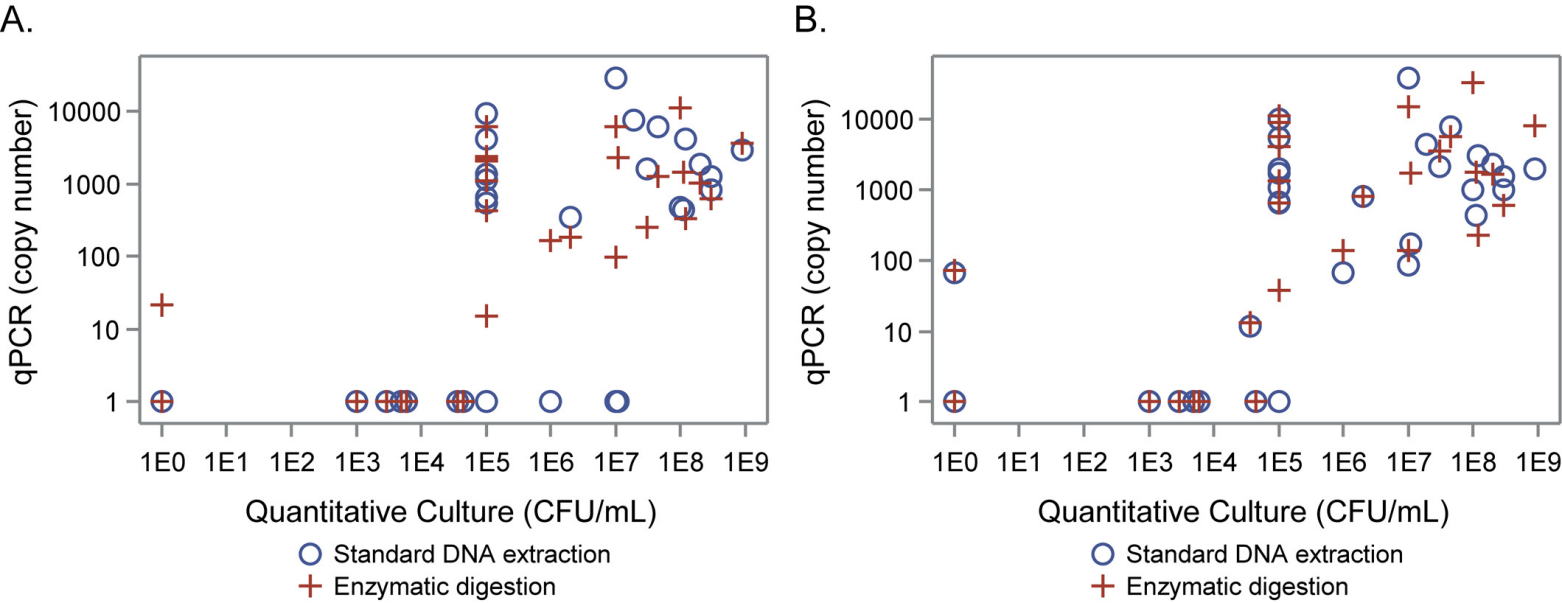
Quantitative Culture vs. qPCR copy number in expectorated sputum samples. qPCR copy numbers for 16S rRNA (A) and femA qPCR (B) assays with (circles) and without enzymatic digestion (plus symbol) plotted vs. CFU/mL from quantitative culture of expectorated sputum samples. Both axes are plotted on a logarithmic scale.

The 16S rRNA qPCR assay with enzymatic digestion and the femA assay regardless of enzymatic digestion detected *S*. *aureus* from all samples with quantities greater than 1 x 10^5^ CFU/mL by culture (Fig 4). Conversely, the 16S qPCR rRNA assay without enzymatic digestion only consistently detected *S*. *aureus* in quantities greater than 1 x 10^7^ CFU/mL by culture. In the 29 ES samples with *S*. *aureus* detected by culture, the correlations between the quantitative culture results (CFU/ml) and the qPCR results (copy number, both 16S and femA with and without enzymatic digestion) ranged from 0.54 to 0.57, p < 0.01 for all. The same comparison for the sequencing data resulted in a correlation of 0.46 for standard DNA extraction and 0.50 with enzymatic digestion (p = 0.01 for both).

**Fig 5:**
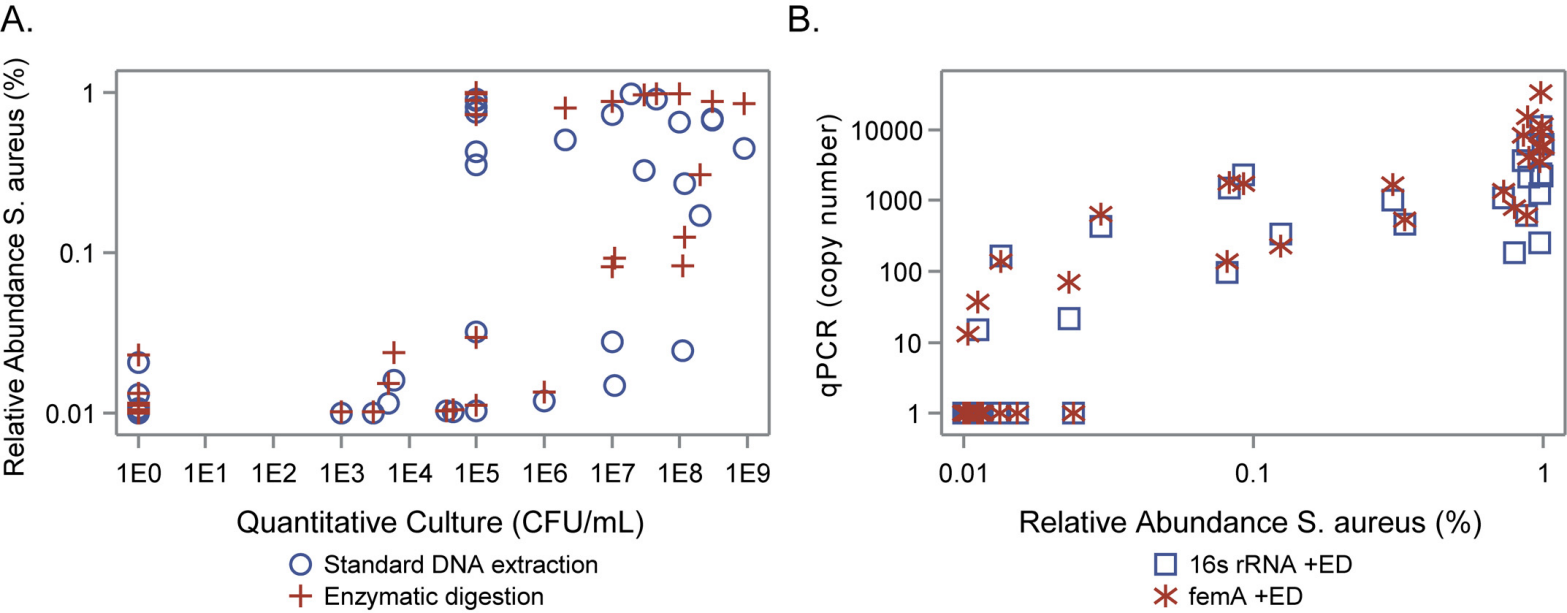
Comparison of species specific data for *S*. *aureus* in ES samples. (A) Quantitative culture versus sequencing. Relative abundance determined with sequencing plotted versus CFU/mL from quantitative culture. (B) Sequencing versus femA qPCR. femA copy number versus relative abundance of *S*. *aureus*.

The relative abundance measured by sequencing was correlated with both the quantitative culture results and copy numbers detected with qPCR (Fig 5). Sequencing with and without enzymatic digestion consistently detected *Staphylococcus* when present in quantities greater than 1 x 10^3^ CFU/mL by culture. Sequencing also identified *Staphylococcus* in 22 culture negative samples all of which had relative abundance levels of *Staphylococcus* less than 0.3% (Fig 5). There were also 20 culture positive samples with relative abundance levels less than 0.1%. The majority of these were OP samples. The 4 ES samples had quantitative culture growth above 10^3^ CFU/mL. Based on our quantitative comparison of culture and sequencing, we estimate that relative abundance levels less than 0.1% correspond to quantitative culture levels at or below 1 x 10^3^ CFU/mL (Fig 5).

### Results of Blast analysis of Staphylococcus

To ensure the sequence comparison to culture was appropriate, we further analyzed the *Staphylococcus* sequences extracted without enzymatic digestion to obtain species level taxonomy where possible. *Staphylococcus* sequences in this study were predominated by *S*. *aureus* (90%). All samples with *Staphylococcus* sequences contained sequence identified as *S*. *aureus*; sensitivity and specificity compared to culture did not change with species level information.

## Discussion

Sequencing demonstrated improved sensitivity and lower detection limits compared to the qPCR assays regardless of inclusion of enzymatic digestion in the DNA extraction protocol. Furthermore, the enzymatic digestion process improved the sensitivity of sequencing with the greatest gain seen with the OP samples. Optimal sensitivity (100%) was achieved with sequencing with enzymatic digestion in ES samples. Sequencing detected *S*. *aureus* in multiple culture negative samples but not in negative controls and thus may even be more sensitive than culture. With a detection limit below 1 x 10^3^ CFU/mL (the limit for culture), sequencing has the potential to identify additional infections. The clinical relevance of the presence of *S*. *aureus* in densities below 1 x 10^3^ requires further investigation. Many of the practical applications of the qPCR assays were overcome with the use of the sequencing. The main limitation of the sequencing as currently performed is its difficulty to distinguish *S*. *aureus* from other *staphylococcal* species. As with the qPCR assays employed in this study, sequencing is also unable to distinguish MSSA and MRSA. The mecA qPCR assay is a molecular assay that has the potential to distinguish between MSSA and MRSA.

From this study, the sensitivity of the qPCR assays does not appear sufficient to be clinically applicable. While the sensitivity of the femA assay is better compared to the 16S rRNA assay, the femA assay is still not as sensitive as culture. In particular, it is limited in its ability to detect *S*. *aureus* in oropharyngeal swab samples. We suspect that the OP swabs have lower bacterial biomass, based on lower DNA yield, which is why they behave differently than sputum samples. The addition of enzymatic digestion did appear to improve detection in the OP samples, along with increasing DNA yield. There are multiple ways in which the addition of enzymatic digestion may have increased the DNA yield. First, the goal of the enzymatic digestion was to specifically aid in the digestion of the *S*. *aureus* cell wall. In this way, the enzymatic digestion may help free the DNA for detection, particularly where bacterial biomass is lower. Additionally, the DNA extraction method with enzymatic digestion also included an additional freeze-thaw cycle when the repeat extractions were performed. The freeze-thaw cycle may also increase the amount of DNA extracted irrespective of enzymatic digestion, but we did not test this step. While the rapid quantitative detection of *S*. *aureus* using qPCR in patients with CF would be useful in guiding immediate antibiotic therapy before the culture results can be obtained, the low sensitivity of this assay limits its possible use clinically.

Methods relying on DNA sequencing techniques are becoming less expensive and increasingly prevalent in the clinical environment. 16S rRNA gene sequencing has recently been used as an alternative method for molecular detection. 16S rRNA gene sequencing is useful because the 16S rRNA gene is present in all bacteria, is well conserved, and is large enough for informatics purposes [27]. Thus, sequencing could be reasonably applied in the clinical setting, but is currently limited by cost and time to results. However, as the cost and time to results of sequencing continue to decrease, sequencing may soon be a practical alternative to culture in the clinical setting. Similar to previous work, our results demonstrated the detection of *S*. *aureus* in culture negative samples, suggesting that sequencing has the potential to be more sensitive than culture [28]. Furthermore, it has the potential to provide the advantages of earlier targeting of antibiotic therapy and the ability to track the resolution of infections.

The 16S rRNA qPCR assay has been shown to be able to successfully identify multiple types of bacteria in multiple sample types [9]. Our group previously demonstrated that qPCR amplification of the 16 rRNA gene can be used to reliably detect *P*. *aeruginosa* and *H*. *influenzae* in patients with cystic fibrosis [6]. In these studies, qPCR demonstrated increased sensitivity compared to culture for *P*. *aeruginosa* (75%), and *H*. *influenza* (75%) but similar to our currently findings, decreased sensitivity compared to culture for *S*. *aureus* (35%) [6]. Francois and colleagues demonstrated the effectiveness of an alternative assay for *S*. *aureus* identification that relies on the amplification of the femA gene [11]. The sensitivity of the qPCR assay in these samples was comparable to culture in nasal, inguinal, and wound samples in hospitalized patients at risk for *S*. *aureus* infection (100%) [11]. However, the femA assay did not improve the detection of *S*. *aureus* in our samples from children with CF, suggesting that CF may provide unique challenges in the molecular identification of *S*. *aureus*. *S*. *aureus* is a prominent pathogen in patients with CF, so it would be expected to contribute significantly to the bacterial community [3]. The standard DNA extraction method may account for the decrease in sensitivity in CF samples [7]. In fact, the addition of this method did significantly improve the sensitivity of multiple molecular methods in our studies.

Methicillin resistance is an important factor in targeting antibiotic therapy in the clinical setting. The *mecA* gene is the gold standard for detecting methicillin resistance and can be used in conjunction with the femA assay to distinguish between MSSA and MRSA. The *mecA* assay demonstrates limitations as well. Francois and co-workers demonstrated a high number of false positive results with the mecA gene [11]. The *mecA* gene also occurs in coagulase negative *staphylococci* (CNS). However, MRSA can be distinguished from CNS with the combination of the femA, femB, and mecA assays [29]. Another potential limitation is that there is evidence that detecting the mecA gene decreases with storage at -80°C over two years [30]. Although distinguishing between MSSA and MRSA is very important clinically, the main aim of the study was the detection of *S*. *aureus*. The tests to assess resistance merit further study.

Our study is not without limitations. First, our sample size is relatively low, and larger studies may be required to further evaluate the effectiveness of these molecular methods. Additionally, *S*. *aureus* was detected in a large proportion of samples by sequencing. However, it is currently unclear whether the small relative abundance seen in many sample is clinically relevant. Finally, it is unclear how the enzymatic digestion step affected the detection of other organisms.

## Conclusion

Our work suggests that sequencing has a detection limit that is low enough to provide useful clinical information in the setting of *S*. *aureus* infection in patients with CF. Based on our studies, sequencing can detect samples with lower quantities compared to cultures and just above the lowest reported values by culture. Quantitative PCR may be useful for rapidly detecting and quantifying *S*. *aureus*, but requires appropriate DNA extraction. However, sensitivity is lower than sequencing, particularly for OP swabs. Future work should also focus on the ability to differentiate between MSSA and MRSA. Sequencing could be a useful tool to track the effectiveness of antibiotic therapy and resolution of infection in the clinical setting.

## Supporting Information

S1 Table Aggregate qPCR and Sequence Data by Sample (XLSX)
